# Metagenomic analysis of a sample from a patient with respiratory tract infection reveals the presence of a γ-papillomavirus

**DOI:** 10.3389/fmicb.2014.00347

**Published:** 2014-07-08

**Authors:** Marta Canuti, Martin Deijs, Seyed M. Jazaeri Farsani, Melle Holwerda, Maarten F. Jebbink, Michel de Vries, Saskia van Vugt, Curt Brugman, Theo Verheij, Christine Lammens, Herman Goossens, Katherine Loens, Margareta Ieven, Lia van der Hoek

**Affiliations:** ^1^Laboratory of Experimental Virology, Department of Medical Microbiology, Center for Infection and Immunity Amsterdam, Academic Medical Center, University of AmsterdamAmsterdam, Netherlands; ^2^CBS-KNAW Fungal Biodiversity CenterUtrecht, Netherlands; ^3^Julius Center for Health Sciences and Primary Care, University Medical Center UtrechtUtrecht, Netherlands; ^4^Department of Medical Microbiology, Vaccine and Infectious Disease Institute, Universiteit Antwerpen–University Hospital AntwerpAntwerp, Belgium

**Keywords:** virus discovery, papillomavirus, respiratory tract infection, VIDISCA-454, GRACE

## Abstract

Previously unknown or unexpected pathogens may be responsible for that proportion of respiratory diseases in which a causative agent cannot be identified. The application of broad-spectrum, sequence independent virus discovery techniques may be useful to reduce this proportion and widen our knowledge about respiratory pathogens. Thanks to the availability of high-throughput sequencing (HTS) technology, it became today possible to detect viruses which are present at a very low load, but the clinical relevance of those viruses must be investigated. In this study we used VIDISCA-454, a restriction enzyme based virus discovery method that utilizes Roche 454 HTS system, on a nasal swab collected from a subject with respiratory complaints. A γ-papillomavirus was detected (complete genome: 7142 bp) and its role in disease was investigated. Respiratory samples collected both during the acute phase of the illness and 2 weeks after full recovery contained the virus. The patient presented antibodies directed against the virus but there was no difference between IgG levels in blood samples collected during the acute phase and 2 weeks after full recovery. We therefore concluded that the detected γ-papillomavirus is unlikely to be the causative agent of the respiratory complaints and its presence in the nose of the patient is not related to the disease. Although HTS based virus discovery techniques proved their great potential as a tool to clarify the etiology of some infectious diseases, the obtained information must be subjected to cautious interpretations. This study underlines the crucial importance of performing careful investigations on viruses identified when applying sensitive virus discovery techniques, since the mere identification of a virus and its presence in a clinical sample are not satisfactory proofs to establish a causative link with a disease.

## INTRODUCTION

Acute respiratory tract infections (ARTIs) can be caused by a wide variety of pathogens, among which viruses and bacteria are the main agents involved. Still, even with the sensitive diagnostic assays employed nowadays, a substantial amount of respiratory diseases cannot be attributed to any of the known commonly involved microorganisms (Tsolia et al., 2004; van de Pol et al., 2006; Regamey et al., 2008).

The existence of previously unknown or unexpected respiratory pathogens can be postulated as an explanation of this phenomenon. Furthermore, the pool of human respiratory viruses keeps growing because of the continuous introduction of “novel” pathogenic viruses from the animal reservoir (Neumann et al., 2009; Memish et al., 2014). These viruses need to be quickly identified and characterized, since they might represent a significant public health concern (Peiris et al., 2004). In both cases the application of broad-spectrum virus discovery techniques can be useful to detect previously unrecognized or emerging pathogens, and the introduction in the market of high-throughput sequencing (HTS) methodologies considerably improved the efficacy of such methods (Mokili et al., 2012).

One of these methods is VIDISCA-454, a restriction enzyme based virus discovery technique which was developed in our laboratory and utilizes Roche-454 as HTS method (de Vries et al., 2011, 2012). This technique provides the broad-spectrum approach needed to identify novel or unexpected viruses (Oude Munnink et al., 2014). In this study, we used VIDISCA-454 to investigate a nasal swab – that tested negative to the principal respiratory pathogens – collected from a patient with respiratory complaints to determine whether an unknown virus could be the cause of the disease.

Among the obtained sequence reads an unexpected virus was found, a papillomavirus. Papillomaviruses carry a circular double stranded DNA genome, are non-enveloped and are known for their capacity to induce warts and benign lesions of mucous membranes (condylomas; Howley and Lowy, 2007). Some human papillomaviruses (HPVs) can cause cancers, of which cervical cancer is the most notorious (Bosch et al., 1995). HPVs are not known as causative agents of respiratory infections, although some studies report the presence of different HPVs types in the respiratory tract of infants with respiratory diseases but also in healthy adults (Kurose et al., 2004; Martinelli et al., 2012; Forslund et al., 2013; Mokili et al., 2013). HPVs are also involved in recurrent respiratory papillomatosis (RRP), a disease characterized by growth of tumors around the vocal cords and in the larynx (Mammas et al., 2014). The pathogenic role of HPVs seems to be genus/type specific: cervical cancer and RRP are induced by α-HPVs (particularly types 16, 18, in cervical cancer, type 11 and 6 in RRP), while γ-HPVs are known to be causative agents of warts or skin lesions. Furthermore, several HPVs, especially from the δ- and γ-genus, can be detected on healthy normal skin (Astori et al., 1998; Antonsson et al., 2000; Li et al., 2012).

Since the high sensitivity of HTS based virus discovery methods makes it possible to identify viruses which are present in a clinical sample at a very low concentration, questions about the clinical relevance of those microorganisms might rise. The scope of this study was to determine whether a correlation could be demonstrated between the γ-HPV – identified with VIDISCA-454 – and the clinical respiratory manifestations of the patient.

## MATERIALS AND METHODS

### PATIENT INFORMATION

The sample was collected from a 64-year-old heavy smoking male patient (defined from now on as the index patient) during a visit to his local general practitioner where he reported symptoms of a respiratory tract infection. The onset of symptoms was 2 days before visiting his general practitioner. Symptoms included cough, phlegm production, shortness of breath, wheeze, coryza, fever, chest pain, muscle aching, and headache. During this first visit respiratory specimens (nasopharyngeal flocked swabs: NPFS, Copan) from each nostril (one in universal transport medium: UTM – the other in skimmed milk) and serum were collected (Visit 1: V1). The patient was part of the GRACE study, a randomized antibiotics placebo-controlled double-blind trial (), and received antibiotics (amoxicillin). A chest X-ray was performed and the patient kept a diary to monitor the course of symptoms and their severity. Fourteen days after onset of symptoms the patient fully recovered. Four weeks after the first visit the patient underwent a check up visit and again specimens from each nostril and serum were collected (Visit 2: V2). The NPFS of the first and second visit were screened for the presence of known respiratory pathogens associated with ARTI: adenovirus, respiratory syncytial virus, human metapneumovirus, influenza A and B, parainfluenza viruses 1–4, human bocavirus, human coronaviruses (229E, OC43, and NL63), human rhinovirus, polyomaviruses WU and KI, *Mycoplasma pneumoniae*, *Chlamydophila pneumoniae*, *Bordetella pertussis*, *Legionella pneumophila*, *Streptococcus pneumoniae,* and *Haemophilus* spp (Loens et al., 2012). All diagnostics remained negative.

### ETHICAL APPROVAL

Ethics review committee of Cardiff and Southampton (UK) approved the study and written informed consent was provided by the participants (Loens et al., 2012).

### VIRUS DISCOVERY: VIDISCA-454

VIDISCA-454 (virus discovery cDNA-AFLP, amplified fragment-length polymorphism combined with Roche 454 high-throughput sequencing) was performed with 110 μl of sample resuspended in UTM as previously described (de Vries et al., 2011). Obtained reads were compared to known sequences present in the non-redundant database using the BLAST tool (Altschul et al., 1990).

### FULL GENOME SEQUENCING

The γ-papillomavirus fragments identified after performing VIDISCA-454 were used as template for primer design and PCRs were performed using the Expand Long Template PCR system (Roche). The complete genome was obtained by sequencing two large amplified fragments which were overlapping on both sides for at least 400 nucleotides. Fragments were cloned into a XL-TOPO-kit (invitrogen), and sequenced (BigDye®; Terminator v1.1 Cycle Sequencing Kit, Applied Biosystems). Primer sequences used in this study are available on request.

### PROTEIN EXPRESSION

The full late gene L1 (coding for the major capsid protein) was amplified with the Expand Long Template PCR system (Roche), cloned into pET100D expression vector (Invitrogen), and transformed into chemically competent Escherichia coli BL21-derived strain Rosetta 2 (Novagen). Overnight cultures of the transformed bacteria were inoculated into Luria broth medium, supplemented with 1% glucose, carbenicillin (10 μg/ml), and chloramphenicol (17.5 μg/ml). Cultures were grown to the exponential phase prior to induction with 0.5 mM isopropyl-β-D-thiogalactopyranoside (IPTG) for 5 h. Samples were collected every hour to monitor the L1-protein production. Attempts to purify the protein via Ni-NTA resin (Qiagen) failed, probably due to the formation of aggregates that shield the HIS-Tags; therefore the crude harvest was used to determine the reactivity of V1 and V2 serum on Western blot.

### SDS PAGE AND WESTERN BLOT

Protein production on SDS-Page was visualized by coomassie staining and SDS-page gels were blotted onto PVDF (Millipore) membranes. The blots were incubated with 1:600 dilutions of either V1 or V2 serum, and with a 1:1000 dilution of a rabbit anti HIS-tag, followed by incubation with anti-human IRDye800CW (1:5000, Jackson Immunoresearch) and anti-rabbit DyLight649 (1:5000). Signals were measured via the Odyssey infrared imaging system (LI-COR). The intensity of the signals was normalized with the anti-HIS signal to correct for difference in protein concentrations.

### SCREENING BY REAL TIME PCR

Real time PCRs were performed with the Platinum®; qPCR-kit (Invitrogen) on an ABI PRISM 7000 sequence detection system of Applied biosystems according to the manufacturer’s protocol, using the following primers and probe: RTForward-540–570: 5^′^-CATACCCTAACGAAGAGGTAGCAGAC-3^′^, RTReverse-675–700: 5^′^-TGGCGGGCAACTGCCTTTATCTAG-3^′^, RTProbe-620–650: 5^′^ FAM- GGGCTGTATTCCGCCAACTGGTGAATATTGGGATG -TAMRA 3^′^.

Screening was performed in duplicate on 54 respiratory samples collected from patients with respiratory complaints involved in the GRACE study (Loens et al., 2012; 25 of which from the same city of the index patient) and only samples having detectable virus in both tests were considered positive.

## RESULTS

A NPFS was collected from a 64 years old man with respiratory symptoms and the diagnostics for the most common respiratory viruses and bacteria remained negative. After performing virus discovery a total of 13,452 sequence reads were obtained and compared with all known sequences in GenBank (NCBI). Among these, 10 sequences showed significant identity to HPVs. By genome walking the entire sequence of the virus (named HPV isolate A2619) was determined: the viral genome is 7142 bp in size, circular and 99% identical to the recently described HPV-KC5 isolate (GenBank accession number: JX444073; Li et al., 2012).

### CORRELATION BETWEEN HPV-A2619 AND THE RESPIRATORY DISEASE

The γ-HPVs are not known as respiratory pathogens and their role in any disease is still uncertain. Therefore, we considered it important to investigate the possible correlation between this virus and the respiratory symptoms of the patient: specific diagnostic tests were developed and applied.

Since the patient fully recovered after 14 days of illness, an increase of the specific antibody response to the responsible pathogen was expected, along with viral clearance. We examined the clearance of the virus with an HPV-A2619 strain specific real time PCR targeting the L1 gene. The virus was detected in the NPFS of both nostrils at V1, but also in the NPFS of both nostrils after the patient recovered (V2). The viral load in all samples was very low, between 2E^3^ and 2E^4^ copies/mL, and the Ct values were similar for all samples (V1-right nostril 38.93, V1-left nostril 40.60, V2-right nostril 42.90, and V2-left nostril 37.47).

**FIGURE 1:**
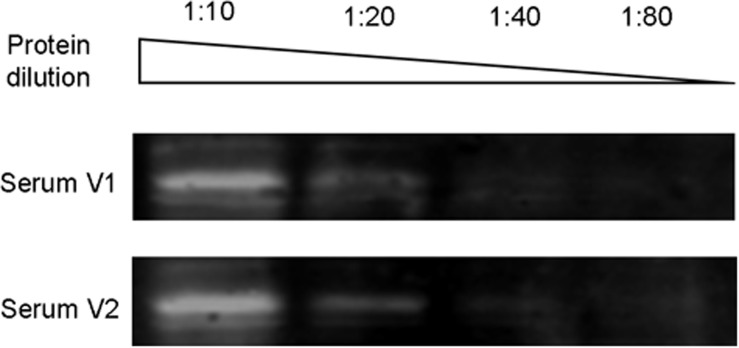
**No increase in antibody levels directed to γ-papillomavirus A2619.** Visit 1 (V1) and Visit 2 (V2) sera were diluted 1:600 and incubated on Western blot containing a dilution series of L1 protein.

Furthermore, western blot analysis was used to detect antibodies directed against the L1 capsid protein in serum samples collected at the same time as the 2 respiratory samples. A poor recognition of the protein was observed and no difference between V1 or V2 serum was noticed: the two samples had similar intensity ratios (10.78% for V1 serum; 10.76% for V2 serum;**Figure 1**).

### SCREENING

Respiratory samples of 54 other patients from the GRACE study with respiratory tract infection were used for prevalence inference. Of these, 25 were from the same geographical area of the index patient and 39 from other European cities. Two patients (3.7%) were positive for HPV-A2619. In both patients also a low viral copy number was detected (between 2E^3^ and 2E^4^ copies/ml). Both patients came from the same city in the UK as the indexcase.

## DISCUSSION

### γ-HPVs AND RESPIRATORY INFECTIONS

Not much is known about γ-papillomaviruses (γ-HPVs). Occasionally γ-HPVs have been related to skin warts or keratotic lesions of immunosuppressed organ transplant recipients (e.g., HPV-4; Köhler et al., 2011), but these viruses can also be detected on healthy skin (Astori et al., 1998; Antonsson et al., 2000; Li et al., 2012), indicating that they might not be pathogenic in healthy individuals or only when specific conditions are fulfilled. We identified a γ-HPV in the respiratory tract of a patient with symptoms of a respiratory tract infection but, since the virus was still detectable after full recovery and no rise in antibodies was observed, we concluded that the virus was not the causative agent of the respiratory disease of this patient. Careful inspection of the chest X-ray also did not reveal any cancer-related abnormalities within the lungs; furthermore also no skin warts or abnormalities on the skin were noted. It is therefore most likely that HPV-A2619, which we describe here, is a harmless and ubiquitous γ-HPV which can be present in nostrils.

HPV-A2619 resulted 99% identical to the previously reported HPV-KC5 which was isolated from normal skin of healthy individuals in a rural area in China (Li et al., 2012), suggesting that the virus is not geographically restricted to one location, as our screening results might have indicated. Furthermore, it might be argued that the virus was introduced in the respiratory tract after contact with contaminated skin during nasal swabbing, but the fact that the virus was persistently present in each nostril at two time points suggests that virus is genuinely present in the nose, as well as on normal skin.

In literature it is described that some types of HPV can cause human papillomatosis, lesions in the respiratory tract that need to be surgically removed, and in most cases it is a recurring problem and multiple surgical treatments are needed (Mammas et al., 2014). The patient described here did not have a history of human papillomatosis, and the symptoms lasted only for 14 days, thus no indication could be found that human papillomatosis was involved. It has also been suggested that HPV, particularly type 16, can play a role in oropharyngeal cancers (D’Souza et al., 2007); however, the clinical data from this patient gave no evidences of tumor.

We therefore concluded that HPV-A2619 does not have a specific pathogenic role in a detectable disease and our results are in agreement with other studies which reported the presence of HPVs in the respiratory tract (Kurose et al., 2004; Martinelli et al., 2012; Forslund et al., 2013; Mokili et al., 2013).

### SENSITIVE METAGENOMIC METHODS AND PATHOGEN DISCOVERY

Thanks to the advances in molecular biology – especially the development of next generation HTS methods – virus discovery techniques became extremely sensitive and VIDISCA-454 (the method developed in our laboratory) is not an exception (de Vries et al., 2011, 2012). The load of HPV-A2619 was determined by real time PCR, and only 200 copies per 100 μl (input in VIDISCA) were measured. This sensitivity is remarkable and might be related to the nature of the virus. HPVs carry a double stranded DNA genome and their detection by VIDISCA-454 does not depend on reverse transcription and second strand synthesis, procedures which decrease the detection potential of RNA viruses.

With state of the art HTS based virus discovery techniques and the possibility to obtain such a huge number of sequences from a clinical sample, the identification of novel viruses became very effective, and nowadays the detection of low load viruses is not uncommon since minority nucleic acids can be efficiently sequenced (Mokili et al., 2012; Cotten et al., 2014). These methods are exclusively based on random sequencing of nucleic acids and they do not provide further information beyond the recognition of viral genetic material in a clinical sample, which does not necessarily correlate with the presence of a pathogen in the sample. In fact, some of these sequences might be derived from contaminating viruses introduced during the sampling or the sample processing procedures (Naccache et al., 2013). It is therefore important to confirm the presence of the recognized viruses in the original sample and to investigate the clinical relevance of every discovery to avoid drawing false conclusions.

With the development of HTS techniques the experimental time needed to discover novel viruses has dramatically reduced. However, targeted experiments and careful evaluation of all available data are essential to assess the relevance of every finding and to determine the role of every identified virus, since – as we show here – the mere identification of a virus is not sufficient to prove its involvement in a disease. This is especially true when dealing with body parts which are contiguous with the external environment – like the respiratory tract – and therefore more subjected to the presence of bystander or non-host-specificmicroorganisms.

## AUTHOR CONTRIBUTIONS

Marta Canuti and Martin Deijs performed the experiments, participated to study design and wrote the paper. Seyed M. Jazaeri Farsani, Melle Holwerda, Maarten F. Jebbink, and Michel de Vries performed the experiments. Saskia van Vugt, Curt Brugman, Theo Verheij, Christine Lammens, Herman Goossens, Katherine Loens, and Margareta Ieven provided the clinical samples and participated to the study design. Lia van der Hoek participated to the study design, supervised the study and helped with the manuscript preparation. All authors critically revised the manuscript and approved the final version.

## Conflict of Interest Statement

The authors declare that the research was conducted in the absence of any commercial or financial relationships that could be construed as a potential conflict of interest.
